# Temperature-dependence of the influence of the position-2-methyl group on the structure-directing effect of piperazine in the synthesis of open-framework aluminophosphates

**DOI:** 10.1038/srep22019

**Published:** 2016-02-25

**Authors:** Pai Huang, Jun Xu, Guodong Qi, Feng Deng, Ruren Xu, Wenfu Yan

**Affiliations:** 1 State Key Laboratory of Inorganic Synthesis and Preparative Chemistry, College of Chemistry, Jilin University, 2699 Qianjin Street, Changchun 130012, P. R. China; 2 State Key Laboratory of Magnetic Resonance and Atomic and Molecular Physics, Wuhan Institute of Physics and Mathematics, The Chinese Academy of Sciences, Wuhan 430071, P. R. China; 3 Institute of Modern Agriculture, Jilin Economic Management Cadre College, 429 Guigu Street, Changchun 130012, P. R. China

## Abstract

The temperature-dependence of the influence of the position-2-methyl group on the structure-directing effect of piperazine in the synthesis of open-framework aluminophosphates was investigated. By heating the initial mixture with the composition of Al_2_O_3_:1.5 P_2_O_5_:R:125 H_2_O at 160 °C, where R is the structure-directing agent of 2-methylpiperazine or piperazine, layered aluminophosphates APMeP150 (R = 2-methylpiperazine) and AP2pip (R = piperazine) were obtained. When the same initial reaction mixture was heated at 190 °C, layered aluminophosphates APMeP200 (R = 2-methylpiperazine) and AP2pip (R = piperazine) were crystallized. APMeP200 and AP2pip have the same inorganic sheet topology. We investigated the crystallization processes of the four open-framework aluminophosphates and found that increasing the heating temperature slowed the crystallization of the initial mixtures. The non-bonding interactions between the inorganic layers of the four open-framework aluminophosphates and the experimental or theoretically generated structure-directing agents were calculated. The possible starting points of the crystallization of the four open-framework aluminophosphates were analysed and compared. The temperature-dependence of the influence of the position-2-methyl group on the structure-directing effect of piperazine revealed that the structure-directing effect, the most important factor in the synthesis of zeolites and related open-framework materials, is determined by multiple factors. The structural parameters of the same compound can be affected by the synthesis conditions.

Zeolites, microporous crystalline aluminosilicates, have been widely used as ion-exchangers in the detergent industry, catalysts in the fluid catalytic cracking (FCC) process, and adsorbents in the drying and purification of natural gas and in the air separation by pressure swing adsorption (PSA) or vacuum pressure swing adsorption (VPSA)^1^. The discovery of aluminophosphate molecular sieves in 1982 increased the structural diversity of the microporous crystalline materials and related open-framework materials^2^. In addition to the sources of the inorganic ions and the solvent, the synthesis of such materials typically requires the utilization of an organic species, which is usually called the “template” or the “structure-directing agent”. To date, hundreds of amines and quaternary ammonium hydroxides have been used. Without a structure-directing agent, the corresponding zeolite or open-framework material cannot be crystallized from the synthetic system. The role the organic amine or quaternary ammonium hydroxide plays in crystallization is called the “template effect” or “structure-directing effect”. The structure-directing effect, especially in the synthesis of the microporous or open-framework aluminophosphates, is explained by the non-bonding interaction between the structure-directing agent and the inorganic open-framework^3^. In the synthesis of microporous or open-framework aluminophosphates, one template can direct more than one structure and one structure can be directed by more than one template. The former is called the “one-template/multiple-structure” phenomenon, whereas the latter is called the “multiple-templates/one-structure” phenomenon. For example, aluminophosphate molecular sieve AlPO_4_-5 (AFI) readily forms with >85 different organic amines, the smallest being isopropylamine (3 carbons) and the largest hexabutyl-1,6-hexanediammonium (30 carbons)^4^. These phenomena have made the explanation of the template effect or structure-directing role of organic species by calculation of their non-bonding interactions with the final inorganic open-framework less convincing. Although significant efforts have been made in investigating the structure-directing effect^5^,^6^,^7^,^8^,^9^,^10^,^11^,^12^,^13^,^14^,^15^,^16^,^17^,^18^,^19^,^20^,^21^,^22^,^23^,^24^,^25^,^26^,^27^,^28^,^29^,^30^,^31^,^32^,^33^,^34^,^35^, the origin of the structure-directing effect and how the structure-directing agent directs the corresponding structure are not fully understood.

In this study, we investigated the structure-directing effect of piperazine (**pip**) and 2-methylpiperazine (**MeP**) at 160 and 190 °C, respectively. The protocol of the experiments is provided in Fig. 1. The influence of the position-2-methyl group on the structure-directing effect of piperazine at 160 and 190 °C and the dependence of the influence on the temperature were examined. The present study may help to correctly understand the nature or origin of the structure-directing effect in the crystallization of microporous materials.

## Results and Discussion

### Overview

Compared with piperazine, 2-methylpiperazine has an extra methyl group at position-2. Thus, investigating the structure-directing effect of piperazine and 2-methylpiperazine allows us to examine the influence of the position-2-methyl group on the structure-directing effect of piperazine. The results presented in this study reveal that the position-2-methyl group influenced the structure-directing effect of piperazine at 160 °C. At 160 °C, piperazine and 2-methylpiperazine directed two layered aluminophosphates with different inorganic sheet topologies. However, the influence of the position-2-methyl group on the structure-directing effect of piperazine disappeared at 190 °C. At 190 °C, piperazine and 2-methylpiperazine directed two layered aluminophosphates with the same inorganic sheet topologies. Therefore, the influence of the position-2-methyl group on the structure-directing effect of piperazine is temperature-dependent. Investigating this influence and the corresponding temperature-dependence may help us understand the nature and origin of the structure-directing effect, a critical factor in the formation of zeolites and related open-framework crystals.

### The structure of APMeP150, AP2pip and APMeP200

According to the literature, the empirical formula of APMeP150 is [Al_6_P_8_O_32_(H_2_O)_2_][C_5_N_2_H_14_]_3_·10H_2_O^36^. The inorganic layer composed of a network of 4-, 6-, and 12-membered rings (MRs), as shown in Fig. 2(a). The 2-methylpiperazine molecules are doubly protonated and located in the interlayer region. There are two and four crystallographically independent Al and P atoms in the unit cell, respectively. One Al atom is connected to four P tetrahedra, the other Al atom is connected to four P tetrahedra and two water molecules, and the P tetrahedra are linked to three Al tetrahedra and possess a terminal P=O bond that points towards the interlayer space.

The empirical formulas of the layered aluminophosphates APMeP200^36^ and AP2pip^37^ are [Al_9_P_12_O_48_][C_5_N_2_H_14_]_4.5_·2.5H_2_O and [Al_9_P_12_O_48_][C_4_N_2_H_12_]_4.5_·5H_2_O, respectively. The topology of the inorganic layer of APMeP200 is the same as that of AP2pip, as shown in Fig. 2(b). The 2-methylpiperazine and piperazine molecules are doubly protonated and are located in the interlayer region. There are nine and twelve crystallographically independent Al and P atoms in the unit cell, respectively. The Al tetrahedra are connected to four P tetrahedra, whereas the P tetrahedra are linked to three Al tetrahedra and possess a terminal P =O bond pointing towards the interlayer space.

### The influence of the position-2-methyl group on the structure-directing effect of piperazine at 160 °C

The molar composition of the initial reaction mixture and the heating temperature (160 °C) for APMeP150 are the same as that for AP2pip, which is denoted as AP2pip-160. The structure-directing agents for APMeP150 and AP2pip-160 were 2-methylpiperazine and piperazine, respectively. Thus, the position-2-methyl group affected the structure-directing effect of piperazine at 160 °C. To understand how and why the position-2-methyl group affects the structure-directing effect of piperazine, we investigated the crystallization processes of APMeP150 and AP2pip-160 with multiple techniques.

#### X-ray diffraction investigation of the crystallization processes of APMeP150 and AP2pip-160

Figure 3 (a) shows the simulated XRD patterns of APMeP150 and the experimental patterns of the solid samples, which were isolated throughout the hydrothermal treatment period. The corresponding XRD patterns for AP2pip-160 are shown in Fig. 3 (b). The concentrations of Al and P and the pH values of the liquid phases in the corresponding products are plotted in Supplementary Fig. S1 and S2, respectively.

In the synthesis of APMeP150, the initial reaction mixture containing 2-methylpiperazine was heated at 160 °C. As shown in Fig. 3(a), no long-range ordering was observed after 30 minutes; however, obvious long-range ordering of APMeP150 was observed after 60 minutes of heating, and highly crystalline and pure APMeP150 was obtained when the initial mixture was heated for 720 minutes. As shown in Fig. S1(a), the concentration of Al in the liquid phase was low and remained nearly constant during the hydrothermal crystallization process, suggesting that the Al source gradually released free Al^3+^ to the liquid phase during the crystallization process. In contrast to the concentration of Al, the concentration of P decreased continuously, indicating that P was gradually solidified into the framework of APMeP150 from the liquid phase. If all P atoms of the P source were left in the liquid phase of the initial reaction mixture, the concentration of P would be 0.6367 mol/L. The ICP analysis showed that the concentration of P in the liquid phase of the initial reaction mixture was 0.3394 mol/L, indicating that approximately half of the P source reacted with the Al source, forming solid amorphous aluminophosphate. When the heating time reached 720 min, at which point the highly crystalline APMeP150 formed, the P concentration in the liquid phase was 0.1845 mol/L, indicating that there was approximately 29 mol% of P left in the liquid phase. Because the Al/P ratios in the initial reaction mixture and the framework of APMeP150 were 6/9 and 6/8, respectively, there was approximately 1/9 (11 mol%) of P would be left in the liquid phase if the Al source was completely consumed. Therefore, the Al source was not completely consumed at this stage, which was confirmed by the NMR analyses of the solid products. During the crystallization, the pH of the liquid phase first decreased and then gradually increased (Fig. S2(a)), indicating that the protons in the liquid phase were first consumed by the 2-methylpiperazine and were then released and consumed again.

When the structure-directing agent in the initial reaction mixture was piperazine instead of 2-methylpiperazine, a longer heating time (120 min) was needed to form the long-range ordering of AP2pip-160, as shown in Fig. 3(b), which suggests that the crystallization of the initial reaction mixture was accelerated when a methyl group existed at the position-2 of piperazine. The long-range ordering prior the formation of crystalline AP2pip-160 was confirmed as piperazinium dihydrogen phosphate (Fig. 3(b)). When the heating time reached 720 min, highly crystalline AP2pip-160 was obtained. Similar to the trends of the changes in the Al and P concentrations and the pH in the crystallization of APMeP150, the concentration of Al in the liquid phase was low and remained nearly constant during the hydrothermal crystallization process, whereas the concentration of P decreased continuously, as shown in Fig. S1(b). When the heating time reached 720 min, at which point the highly crystalline AP2pip-160 was formed, the P concentration in the liquid phase was 0.1039 mol/L, indicating that there was approximately 16 mol% of P left in the liquid phase. As discussed above, approximately 11 mol% of P would be left in the liquid phase if the Al source was completely consumed. Therefore, the Al source was almost completely consumed at this stage, which was confirmed by the NMR analyses of the solid products. Similar to the crystallization process of APMeP150, the pH of the liquid phase first decreased and then gradually increased (Fig. S2(b)).

#### The ^27^Al MAS NMR study of the crystallization processes of APMeP150 and AP2pip-160

To obtain detailed information about the coordination state of Al and P during the crystallization process and their evolution with time, we characterized the products that were isolated throughout the hydrothermal treatment period at 160 °C using solid-state NMR.

Figure 4 shows the ^27^Al MAS NMR spectra of the isolated solid samples that were obtained throughout the hydrothermal treatment period during APMeP150 (a) and AP2pip-160 (b) crystallization. As shown in Fig. 4(a), the signal at 7.8 ppm in the ^27^Al MAS NMR spectrum of the initial mixture containing 2-methylpiperazine is from the aluminium source, and the resonances at 36.6 and −13.9 ppm are from the four- and six-coordinated Al species, respectively. The signal of the four-coordinated Al in the highly crystallized APMeP150 (Fig. 4(a), 720 min sample) is also at 36.6 ppm, indicating that the local environment of the four-coordinated Al in the fragments formed in the initial mixture is the same or very similar to that in the framework of APMeP150. Upon heating (30 min), the shape and position of the signals changed significantly. The signal attributed to the six-coordinated Al (i.e., −13.9 ppm) disappeared completely, whereas the intensity of the signal from the four-coordinated Al (i.e., 36.6 ppm) decreased, suggesting the number of the fragments containing such Al species decreased. The product in this stage was still amorphous (Fig. 3(a)). When the heating reached 60 min, a new signal at 20.9 ppm was observed. This signal was also observed in the spectrum of the highly crystallized product (Fig. 4(a), 90 and 720 min samples). According to the literature, the signal at 20.9 ppm can be attributed to the five-coordinated Al species^38^. There are two crystallographically independent Al atoms in APMeP150, which are both connected to four P tetrahedra. However, 1/6 of the Al atoms are octahedrally coordinated to two additional water molecules with distances of 2.250 and 2.323 Å, which are longer than the other four Al-O bonds (1.67, 1.68, 1.73, 1.73 Å). Thus, the environment of this Al site is different than the environment of the Al site connected to six P tetrahedra via an oxygen bridge, which may produce the signal at 20.9 ppm. When the heating time reached 720 min, a small amount of unreacted Al source still existed in the solid product.

The results in Fig. 4(b) show that the type and distribution of Al sites in the initial mixture containing piperazine are very similar to that containing 2-methylpiperazine, except for the position of the signal from the four-coordinated Al. In the initial mixture containing 2-methylpiperazine, the four-coordinated Al site gives the signal at 36.6 ppm, whereas this position shifts to 36.1 ppm when the 2-methylpiperazine is replaced by piperazine. The signal of the four-coordinated Al in the highly crystallized AP2pip-160 (Fig. 4(b), 720 min sample) is also at 36.1 ppm, indicating that the local environment of the four-coordinated Al in the fragments formed in the initial mixture is the same or very similar to that in the framework of AP2pip-160. Upon heating (30 and 60 min), the Al source was gradually consumed with the formation of six-coordinated Al species. However, the number of four-coordinated sites remained almost constant and then increased. The XRD data show that the long-range ordering of AP2pip-160 was not observed until the heating time reached 120 min. Accordingly, the signal at 36.1 ppm became narrower and intensified. Six-coordinated Al species were observed in this stage but completely disappeared shortly after heating in the presence of 2-methylpiperazine (Fig. 4). Thus, unlike 2-methylpiperazine, piperazine altered the evolution of the six-coordinated Al species. When the heating time reached 720 min, the intense peak at 36.1 ppm was the only signal, and the other signals from the Al source and six-coordinated Al species completely disappeared, indicating that the crystallization process was completed and the highly crystallized product of AP2pip-160 was formed. In the structure of AP2pip-160, there are nine crystallographically independent Al atoms in the asymmetric unit. These crystallographically independent atoms have very similar chemical environments, but slightly differ in bond length and angle, which are insensitive to solid-state NMR detection. As shown in Fig. 4, only one Al peak was observed in the highly crystalline product, which is in accordance with previous reports on this material^23^,^25^,^37^.

As shown in Fig. 4(a,b), the coordination state and the distribution of the Al species in the initial mixture containing 2-methylpiperazine are very similar to that of the Al species in the initial mixture containing piperazine. The local environment of the four-coordinated Al species in both initial mixtures is extremely similar (36.6 ppm for 2-methylpiperazine and 36.1 ppm for piperazine). In addition, the concentration of Al and the pH in the liquid phase for both initial mixtures are also similar. Along with the heating, a significant difference in the evolution of the coordination state of Al species in both initial mixtures was observed. When the structure-directing agent was 2-methylpiperazine, the six-coordinated Al species disappeared in a short time and the number of four-coordinated Al species underwent a decreasing and then increasing process. However, the six-coordinated Al species existed for a long time, and the number of four-coordinated Al species was almost unchanged and then increased when piperazine was used as the structure-directing agent. Compared with piperazine, 2-methylpiperazine has an extra position-2-methyl group. At 160 °C, this extra position-2-methyl group altered the interaction of the doubly protonated piperazine-ring with the surrounding species, which resulted in the change of the crystallization direction of the initial mixture. In addition, even though APMeP150 and AP2pip-160 have completely different inorganic sheet topologies, the local environment of the four-coordinated Al species in both structures is similar. Supplementary Table S1 gives the bond lengths and angles of the four-coordinated Al in APMeP150 and AP2pip-160. The data in Table S1 show that the O-Al-O bond angles in APMeP150 and AP2pip-160 are similar. The differences for the shortest and longest Al-O bond lengths are 2.3% and 2.0%, respectively. Thus, the chemical shift (36.6 ppm) of the signal from the four-coordinated Al sites in APMeP150 is almost the same as that (36.1 ppm) in AP2pip-160.

#### The ^31^P MAS NMR study of the crystallization process of APMeP150 and AP2pip-160

**Figure 1:**
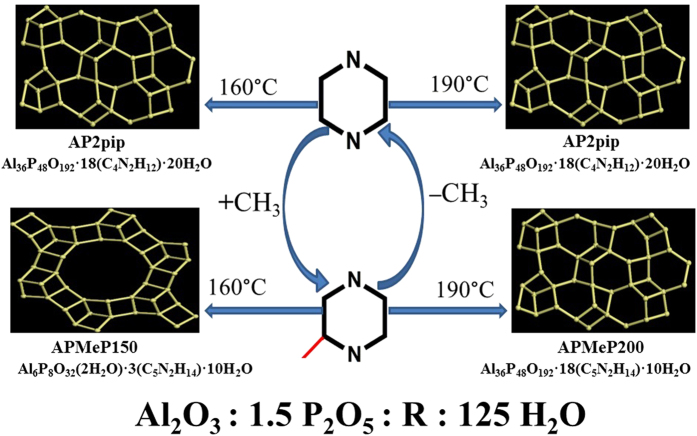
The protocol of the experiments to investigate the temperature-dependence of the influence of the position-2-methyl group on the structure-directing effect of piperazine in the synthesis of open-framework aluminophosphates.

**Figure 2:**
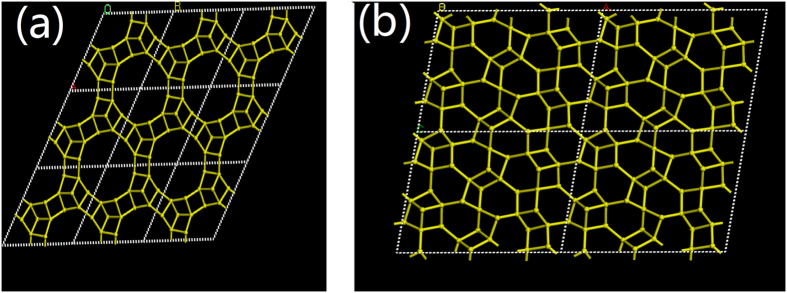
The inorganic layers of APMeP150 (**a**) and APMeP200 and AP2pip (**b**).

**Figure 3:**
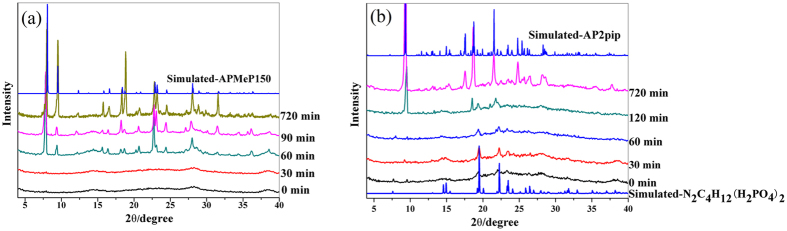
The simulated XRD patterns of APMeP150 (**a**) and AP2pip (**b**) and the experimental patterns of the solid samples isolated throughout the hydrothermal treatment period at 160 °C.

Figure 5 shows the ^31^P MAS NMR spectra of the isolated solid samples that were obtained throughout the hydrothermal treatment period of APMeP150 (a) and AP2pip-160 (b). For the APMeP150 (Fig. 5(a)), a broad signal at ca. –12.2 ppm was observed in the initial mixture containing 2-methylpiperazine, which was assigned to an amorphous aluminophosphate^39^. The coordination number of the P atoms varied from 0 to 4, resulting in a broad signal. Upon heating at 160 °C for 30 minutes, the shape of this broad signal changed slightly. The centre of the signal shifted to –6.4 ppm, and a shoulder peak at –19.4 ppm appeared. This new shoulder peak was also observed in the final crystalline product (720 min sample), indicating that the local environment of the P sites embedded in the fragments formed at this stage is the same or similar to that in the final open-framework. However, the XRD data (Fig. 3(a)) show that the product is amorphous, and the long-range ordering of APMeP150 has not formed at this stage. After extending the heating time to 60 minutes, the intensity of the signal at –19.4 ppm was significantly enhanced, and the broad peak from the P sites in the amorphous aluminophosphate almost disappeared. At this stage, well-crystallized APMeP150 was formed (Fig. 3(a)), confirming that the signal at –19.4 ppm is from the final open-framework of APMeP150. After 720 minutes of heating, at which time the highly crystalline APMeP150 was obtained, an intense asymmetric signal at –19.4 ppm from the APMeP150, a weak shoulder resonance at –12.2 ppm from the residual amorphous aluminophosphate, and a peak at –1.0 ppm from the 2-methylpiperaziniumphosphate were observed. In the structure of APMeP150, there are four crystallographically independent P atoms in the asymmetric unit, which are included in a capped 6-membered ring. As shown in Fig. 1(a), the ratio of the “cap P” and the “corner P” is 1:3. Thus, the intense asymmetric signal at –19.4 ppm can be assigned to the “corner P”, whereas the “cap P” is not resolved.

As shown in Fig. 5(b), the evolution of the coordination state of P in the initial mixture containing piperazine was different from that containing 2-methylpiperazine. The spectrum of the initial mixture shows that the centre of the broad peak from the amorphous aluminophosphate shifted to –11.3 ppm, and the signal from the ordered piperazinium dihydrogen phosphate was observed (4.4 ppm). The XRD data (Fig. 3(b)) show that the long-range ordering of AP2pip-160 was not detected when the heating time reached 60 minutes. Thus, the shape of the spectra of the 0, 30, and 60 minutes samples were similar. After heating for 120 minutes, long-range ordering of the AP2pip-160 appeared, and a shoulder signal at –27.9 ppm was observed. When the heating time reached 720 minutes, at which point the highly crystalline AP2pip-160 was formed (Fig. 3(b)), only a weak resonance centred at 0.8 ppm and a strong resonance that was centred at –27.9 ppm were observed. The former resonance signal was from piperazinium phosphate. According to the single-crystal structure data, there are twelve crystallographically independent P atoms in the asymmetric unit, each of which connected three Al atoms via oxygen atoms and had a terminal P=O bond. Thus, twelve NMR resonances should be observed. However, only one dominant signal at −27.9 ppm appeared, suggesting the overlap of the similar chemical shifts of these P sites.

Comparison of Fig. 5(a) with (b) shows that the coordination state and its distribution of P species in the initial mixture containing 2-methylpiperazine are significantly different to that of the P species in the initial mixture containing piperazine. In contrast to the pH, the evolutions of the concentrations of P in the liquid phase for both initial mixtures are different. Because the molar ratios of the two initial mixtures are identical and the 2-methylpiperazine has an extra position-2-methyl group than piperazine, the difference can be attributed to the presence of the position-2-methyl group.

#### Host-guest interaction in APMeP150 and AP2pip-160

To quantitatively study the influence of the position-2-methyl group on the structure-directing effect of piperazine at 160 °C, we calculated the non-bonding interactions between the SDAs and the inorganic host of APMeP150 and AP2pip-160. Before the calculation was conducted, we performed the dynamic calculations at 433 K (160 °C) for APMeP150 and AP2pip using Material Studio software. After the dynamic calculations, the positions of the atoms in the structures were adjusted slightly to reach a local minimum energy. The final single-point energy of the non-bonding interaction between the inorganic host and the organic guest species was obtained at 0 K. The SDAs include the experimentally determined 2-methylpiperazine and piperazine and the theoretically generated ones by eliminating the methyl group from the 2-methylpiperazine in APMeP150 (denoted as APMeP150 pip from MeP) or by adding a methyl group at position-2 of piperazine in AP2pip-160 (denoted as AP2pip MeP from pip). The non-bonding interactions for these four cases are summarized in Table 1.

The data in Table 1 show that the non-bonding interaction in the experimentally obtained APMeP150 was −81.91 kJ/mol, which was significantly increased to −45.64 kJ/mol if the 2-methylpiperazine was replaced by piperazine generated by eliminating the methyl group from the 2-methylpiperazine. Similarly, the non-bonding interaction in the experimentally obtained AP2pip was −132.50 kJ/mol, and it was significantly increased to −60.59 kJ/mol when the piperazine was replaced by 2-methylpiperazine that was generated by adding a methyl group at position-2 of piperazine in AP2pip-160. Thus, it can be anticipated that when the initial mixture was crystallized at 160 °C, piperazine will direct AP2pip and 2-methylpiperazine will direct APMeP150.

#### The core units of APMeP150 and AP2pip-160

To further investigate, at molecular level, how the position-2-methyl group affects the structure-directing effect of piperazine, we extracted the core units (possible starting points of crystallization) of APMeP150 and AP2pip-160^18^. Figure 6 shows the core units with a close contact of 3.0 Å for APMeP150 (a) and AP2pip-160 (b and c). Because APMeP150 contains one crystallographically independent 2-methylpiperazine molecule and AP2pip-160 contains four and a half crystallographically independent piperazine molecules, the core units of AP2pip-160 are much more complicated than that of APMeP150. For clarity, the core units of the layers above and below in AP2pip-160 are shown in Fig. 6(b,c). The core units of APMeP150 contain one 2-methylpiperazine molecule and two trimers of the aluminophosphate fragment. Strong H-bonds between the trimers and the doubly protonated 2-methylpiperazine molecule are observed. In contrast to the core units of APMeP150, the core units of AP2pip-160 contain four and a half piperazine molecules and more complicated inorganic fragments. The inorganic fragments and the doubly protonated piperazine molecules are arranged in a special configuration via strong H-bonds interactions. Thus, the position-2-methyl group modified the physical and chemical properties of piperazine, which altered the formation of inorganic fragments. In the early stage of crystallization, the core units stabilized by the 2-methylpiperazine are different from the core units stabilized by piperazine. Along with the crystallization, two different structures were obtained, demonstrating the influence of the position-2-methyl group on the structure-directing effect of piperazine.

### The influence of the position-2-methyl group on the structure-directing effect of piperazine at 190 °C

When the initial mixtures producing APMeP150 and AP2pip were heated at 190 °C, the products were layered aluminophosphates APMeP200 ([Al_9_P_12_O_48_][C_5_N_2_H_14_]_4.5_·2.5H_2_O) and AP2pip ([Al_9_P_12_O_48_][C_4_N_2_H_12_]_4.5_·5H_2_O, denoted as AP2pip-190). APMeP200 and AP2pip-190 have the same inorganic layer topology. The doubly protonated 2-methylpiperazine and piperazine have similar positions in the structure of APMeP200 and AP2pip-190. The number of crystalline waters in APMeP200 is half of that in AP2pip-190. Thus, the position-2-methyl group does not affect the structure-directing effect of piperazine at 190 °C. It would be interesting to determine the reason behind this phenomenon.

#### X-ray diffraction investigation of the crystallization processes of APMeP200 and AP2pip-190

Figure 7(a) shows the simulated XRD patterns of APMeP200 and the experimental patterns of the solid samples, which were isolated throughout the hydrothermal treatment period. The corresponding XRD patterns for AP2pip-190 are shown in Fig. 7(b). The concentrations of Al and P and the pH values of the liquid phases in the corresponding products are plotted in Supplementary Fig. S3 and S4, respectively.

As shown in Fig. 7(a), no long-range ordering was observed after 50 minutes; however, obvious long-range ordering of APMeP200 was observed 10 minutes later, and highly crystalline and pure APMeP200 was obtained when the initial mixture was heated for 24 h. Compared with heating at 160 °C, heating at 190 °C slowed the crystallization rate of the initial mixture, indicating that the crystallization kinetics of APMeP200 are significantly different than APMeP150. When 2-methylpiperazine was replaced by piperazine, the appearance of the well-developed, long-range ordering with the same topology was significantly postponed (from 1 h to 3 h, Fig. 7(b)). However, the crystallization process was shortened from 24 h to 6 h (Fig. 7(b)), suggesting that the crystallization kinetics of APMeP200 are significantly different than AP2pip-190, although they have identical inorganic layer topology. After extending the heating time to 12 h, a dense phase of aluminophosphate was obtained. When the initial mixture containing piperazine was heated at 160 °C, well-developed, long-range ordering was observed at 2 h, which is shorter than when heated at 190 °C, indicating that the formation of the stable core units (starting points of crystallization) takes longer at 190 °C than at 160 °C and increasing the heating temperature does not always accelerate the crystallization rate.

As shown in Fig. S3, the concentration of Al in the liquid phase was low and remained nearly constant during the hydrothermal crystallization process, suggesting that the Al source gradually released free Al^3+^ to the liquid phase during the crystallization process. In contrast to the concentration of Al, the concentration of P in the liquid phase during the crystallization of APMeP200 first increased and then decreased continuously. When the crystallization was complete, the concentration of P was 0.1110 mol/L, which was approximately 17 mol% of the total P species. As mentioned above, if the entire Al source was consumed, there would be 11 mol% of P left in the liquid phase. Thus, the Al source was not completely consumed at this stage, which was confirmed by the NMR analyses of the solid products. However, the amount of remaining Al source at 190 °C was less than that at 160 °C. When 2-methylpiperazine was replaced by piperazine, the concentration of P underwent a different evolution. In the initial mixture, approximately half of the P source reacted with the Al source, forming solid amorphous aluminophosphate. Along with the crystallization, the concentration of P in the liquid phase decreased slightly, indicating that the solidification rate accompanied by the ordering is higher than the dissolving rate of amorphous aluminophosphate. After 6 hours of heating, highly crystallized AP2pip-190 was obtained. The concentration of P in the liquid phase was 0.2539 mol/L, which is approximately 40 mol% of the total P species. Thus, Al source was not consumed at this stage, which was confirmed by the NMR analyses of the solid products. Interestingly, the evolution of the pH during the crystallization of APMeP200 and AP2pip-190 was different than that of APmeP150 and AP2pip-160 (Fig. S4). The pH of the liquid phases continuously increased, suggesting that the protons were continuously consumed by the 2-methylpiperazine and piperazine when the heating temperature was 190 °C.

#### The ^27^Al MAS NMR study of the crystallization processes of APMeP200 and AP2pip-190

To obtain detailed information about the coordination state of Al and P during the crystallization process and their evolution with time, we characterized the products of APMeP200 and AP2pip-190 that were isolated throughout the hydrothermal treatment period using solid-state NMR. Figure 8 shows the ^27^Al MAS NMR spectra of the isolated solid samples that were obtained throughout the hydrothermal treatment period during APMeP200 (a) and AP2pip-190 (b) crystallization.

**Figure 4:**
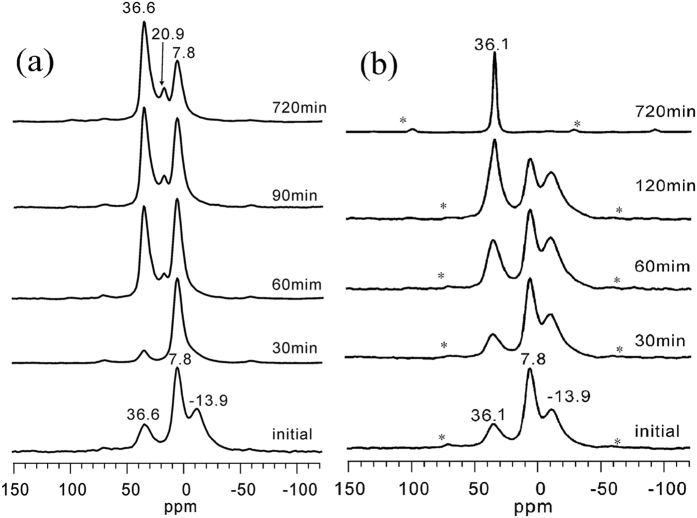
The ^27^Al MAS NMR spectra of the isolated solid samples that were obtained throughout the hydrothermal treatment period of APMeP150 (**a**) and AP2pip-160 (**b**). The asterisks indicate spinning sidebands.

As shown in Fig. 8(a), the signal at 7.8 ppm in the ^27^Al MAS NMR spectrum of the initial mixture containing 2-methylpiperazine is from the aluminium source, and the resonances at 42.6 and –11.3 ppm are from the four- and six-coordinated Al species, respectively. The initial mixture with the same composition produced signals at 36.6 and −13.9 ppm for four- and six-coordinated Al species, respectively, as shown in Fig. 4(a). This difference was unexpected because both initial mixtures were taken from the same source. After re-checking the experimental details, we found that the initial mixture for the crystallization of APMeP200 at 190 °C was stirred 3 h longer than the initial mixture for the crystallization of APMeP150 at 160 °C due to the approximately 3 h loading process for the same amount of the mixture into the Teflon-lined autoclaves. During the loading process, the initial mixture in the large beaker was kept stirring. The initial mixture for the crystallization of APMeP150 was first loaded into a centrifuge tube, and the initial mixture for the crystallization of APMeP200 was loaded into a centrifuge tube at the last stage. Thus, the local environment of the four- and six-coordinated Al species in the initial mixture was significantly changed during the additional 3 h of stirring, and the positions of the corresponding signals were changed to 42.6 ppm from 36.6 ppm for the four-coordinated Al species and to −11.3 ppm from −13.9 ppm for the six-coordinated Al species. The 3 h of additional stirring can be considered an ageing process. Upon heating, the fragments containing the four-coordinated Al species producing the signal at 42.6 ppm and the six-coordinated Al species producing the signal at −11.3 ppm disappeared, and the four-coordinated Al species producing the signal at 39.1 ppm formed. During the crystallization process of the initial mixture, the disappearance of the signal at 42.6 ppm was caused by the decomposition of these fragments or local environment adjustment around the four-coordinated Al species. The present data cannot unambiguously confirm nor exclude either possibility.

Figure 8(b) shows the evolution of the coordination state of Al species during AP2pip-190 crystallization. In addition to the Al source (7.8 ppm), four-coordinated Al species (40.9 ppm) and six-coordinated Al species (−10.4 ppm) were observed in the initial mixture for the crystallization of AP2pip-190 at 190 °C. However, the local environment of the four- and six-coordinated Al species in the initial mixture for the crystallization of AP2pip-190 at 190 °C was different than that of the corresponding four- and six-coordinated Al species in the initial mixture for the crystallization of AP2pip-160 at 160 °C, as shown in Fig. 4(b), even though the two initial mixtures were taken from the same source. In the initial mixture for the crystallization of AP2pip-160, the four- and six-coordinated Al species produced signals at 36.1 and −13.9 ppm, respectively. The difference in the local environment for the Al species with the same coordination state can be attributed to the ageing process (3 h additional stirring) in preparing the initial mixtures. Upon heating, the fragments containing the four-coordinated Al species producing the signal at 40.9 ppm and the six-coordinated Al species producing the signal at −10.4 ppm disappeared, and the four-coordinated Al species producing the signal at 36.4 ppm formed. In the well-crystallized AP2pip-160, the chemical shift of the Al species was 36.1 ppm, indicating that the local environment of the Al species in AP2pip was less affected by the crystallization temperature. However, the local environment might be affected by other factors, such as the type of the structure-directing agent. For example, APMeP200 and AP2pip have identical inorganic topology. However, the Al species in the well-crystallized APMeP200 produced a signal at 39.1 ppm, whereas the Al species in the well-crystallized AP2pip produced a signal at 36.4 or 36.1 ppm. Thus, it is more accurate to say that 2-methylpiperazine and piperazine have the same topological structure-directing effect rather than the same structure-directing effect under such conditions.

#### The ^31^P MAS NMR study of the crystallization process of APMeP200 and AP2pip-190

**Figure 5:**
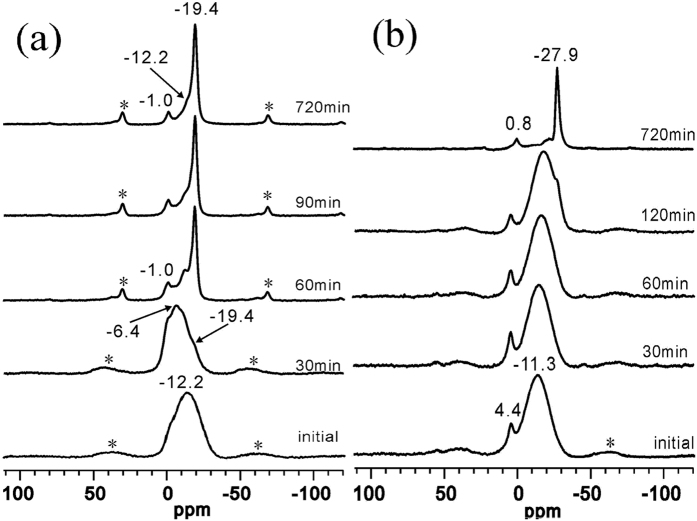
The ^31^P MAS NMR spectra of the isolated solid samples that were obtained throughout the hydrothermal treatment period of APMeP150 (**a**) and AP2pip-160 (**b**). The asterisks indicate spinning sidebands.

Figure 9 shows the ^31^P MAS NMR spectra of the isolated solid samples that were obtained throughout the hydrothermal treatment period of APMeP200 (a) and AP2pip-190 (b). APMeP200, AP2pip-190, and AP2pip-160 have identical inorganic layer topology. AP2pip-190 and AP2pip-160 are the same compounds, crystallized from initial mixtures with the same molar compositions at 190 and 160 °C, respectively. However, the local environment of P sites in AP2pip-190 is significantly different than that in AP2pip-160. The P sites in AP2pip-190 produced a signal at −21.7 ppm, whereas the signal was shifted to −27.9 ppm in AP2pip-160. This result indicates that the structure of AP2pip crystallized at 190 °C is significantly different than that crystallized at 160 °C, even though they have the same topology. In our previous studies, we found that the crystallization pathway of AP2pip depends on the synthesis conditions^31^. The present study confirmed that the structure of AP2pip is also synthesis condition-dependent. In the crystallization of APMeP200, a shoulder peak at −29.7 ppm was observed, which was not observed in the crystallization of APMeP200 from the initial mixture with the same composition at 200 °C^29^. This signal can be assigned to the P sites coordinated by four Al atoms via an oxygen bridge. However, all P sites in APMeP200 were coordinated by three Al atoms via an oxygen bride. Thus, the species containing these P sites must be the intermediates in the crystallization of APMeP200. In the crystallization of AP2pip-160 and AP2pip-190 (compounds possessing the same inorganic layer topology to APMeP200), these intermediates were not formed, as shown in Figs 5(b) and 9(b). Thus, these intermediates were only formed at 190 °C and in the presence of 2-methylpiperazine. These results confirmed our previous observation that the assembly of the small fragments by different crystallization pathways may result in the same compound; however, the structural details of these compounds are different, even though they have the same topology^31^.

#### Host-guest interaction of APMeP200 and AP2pip-190

To quantitatively study the influence of the position-2-methyl group on the structure-directing effect of piperazine at 190 °C, we calculated the non-bonding interactions between the SDAs and the inorganic host of APMeP200 and AP2pip-190. Before the calculation, we performed dynamic calculations at 463 K (190 °C) for APMeP200 and AP2pip using Material Studio software. After the dynamic calculations, the positions of the atoms in the structures were adjusted to reach a local minimum energy. The final single-point energy of the non-bonding interaction between the inorganic host and the organic guest species was obtained at 0 K. The SDAs include the experimentally determined 2-methylpiperazine and piperazine and the theoretically generated ones by adding a methyl group at position-2 of piperazine in AP2pip-190 (denoted as AP2pip MeP from pip). The orientation of the methyl group in the newly generated 2-methylpiperazine is the same as that in the experimentally determined 2-methylpiperazine in APMeP200. The non-bonding interactions for these three cases are summarized in Table 2.

The data in Table 2 show that the non-bonding interactions in two experimentally obtained structures and one with the created structure-directing agents are similar, indicating that 2-methylpiperazine and piperazine have the same structure-directing effect at 190 °C. The initial structure of the “AP2pip MeP from pip” listed in Table 2 is identical to the initial structure of “AP2pip MeP from pip” listed in Table 1. After the dynamic calculations at 160 and 190 °C, the non-bonding interactions changed to −60.59 and −116.17 kJ/mol per T site, respectively. These results show that the influence of the position-2-methyl group on the structure-directing effect of piperazine depended on the crystallization temperature.

#### The core units of APMeP200 and AP2pip

**Figure 6:**
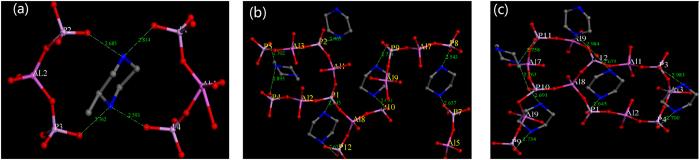
The core units of APMeP150 (**a**) and AP2pip-160 (**b**: above layer, **c**: below layer).

**Figure 7:**
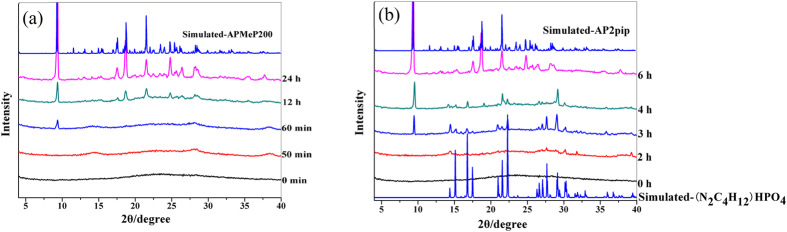
The simulated XRD patterns of APMeP200 (**a**) and AP2pip (**b**) and the experimental patterns of the solid samples isolated throughout the hydrothermal treatment period.

**Figure 8:**
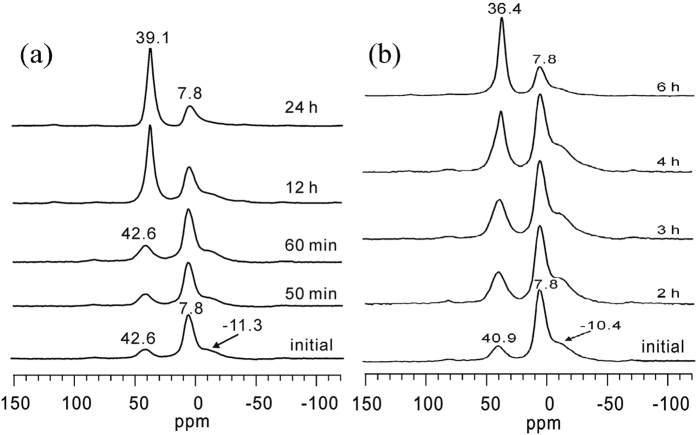
The ^27^Al MAS NMR spectra of the isolated solid samples that were obtained throughout the hydrothermal treatment period of APMeP200 (**a**) and AP2pip-190 (**b**).

**Figure 9:**
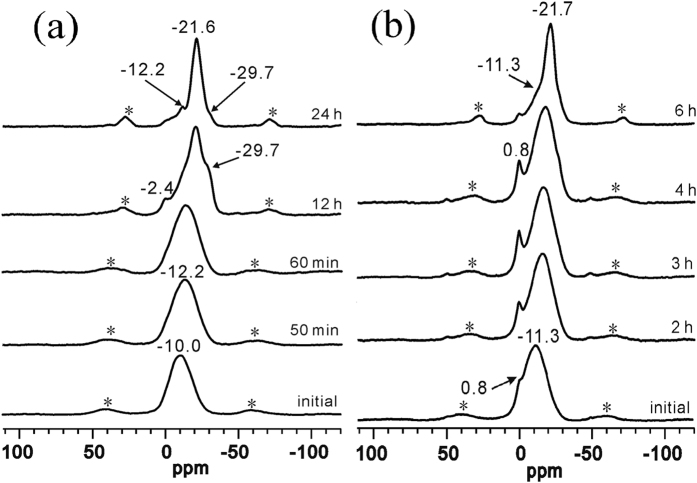
The ^31^P MAS NMR spectra of the isolated solid samples obtained throughout the hydrothermal treatment period of APMeP200 (**a**) and AP2pip-190 (**b**). The asterisks indicate spinning sidebands.

**Figure 10:**
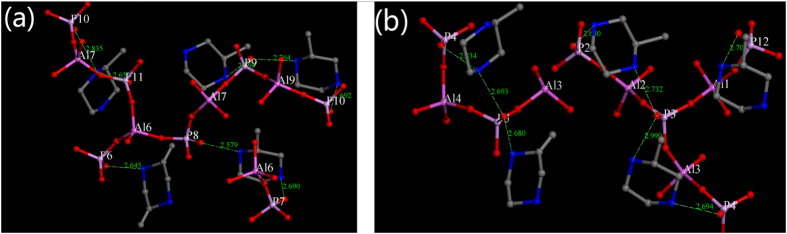
The core unit of APMeP200, (**a**): above layer, (**b**): below layer.

Figure 10 shows the core units with a close contact of 3.0 Å for APMeP200. Similarly to AP2pip, APMeP200 contains four and a half crystallographically independent 2-methylpiperazine molecules. Thus, the core units of APMeP200 are complicated. For clarity, the core units of the layers above and below are shown in Fig. 10(a,b). The corresponding core units of AP2pip are shown in Fig. 6. The results in Figs 6 and 10 show that the core units of APMeP200 are significantly different from those of AP2pip, even though they have the same inorganic sheet topology, which indicates that APMeP200 and AP2pip have different starting points of crystallization. Thus, the “structure-directing effect” described in previously published literature is actually a “topological-structure-directing effect”, as we noted previously^25^. According to this new insight, the topological-structure-directing effect of piperazine is not affected by the position-2-methyl group.

In summary, the initial mixture containing the structure-directing agent of piperazine or 2-methylpiperazine was heated at 160 or 190 °C. Piperazine and 2-methylpiperazine directed two layered aluminophosphates with different topologies at 160 °C, whereas at 190 °C, piperazine and 2-methylpiperazine directed two layered aluminophosphates with identical topologies, showing the temperature-dependence of the influence of the position-2-methyl group on the structure-directing effect of piperazine. Simulation suggested that such temperature-dependence is controlled by the host-guest non-bonding interaction at different heating temperatures. Increasing the reaction temperature does not always accelerate the crystallization rate. The local structure of an open-framework also depends on the synthesis conditions.

## Methods

### Synthesis

The layered aluminophosphates AP2pip, APMeP150, and APMeP200 were obtained from the initial reaction mixture with the molar composition of Al_2_O_3_:1.5 P_2_O_5_:R:125 H_2_O, where R is the structure-directing agent of 2-methylpiperazine or piperazine. If the structure-directing agent was 2-methylpiperazine, APMeP150 and APMeP200 were crystallized from the initial reaction mixture at 160 and 190 °C for 12 h, respectively. If the structure-directing agent was piperazine, AP2pip was the only product, whether the initial reaction mixture was heated at 160 or 190 °C for 12 h. The procedure used for the preparation of the initial reaction mixture was as follows: 6.90 g of 85 wt% phosphoric acid (85 wt% H_3_PO_4_) was stirred with 90 mL of water, and 2.80 g of boehmite (Catapal B, 72.7% Al_2_O_3_, Sasol) was added to the mixture. After the mixture was thoroughly stirred for 1 h, 2.00 g of 2-methylpiperazine or 3.80 g piperazine hexahydrate was added with continuous stirring. The mixture was stirred for 1 h at ambient temperature to ensure homogeneity. The same amount of mixture was loaded into several Teflon-lined autoclaves using a syringe while stirring. The autoclaves were then placed in a pre-heated oven at 160 °C or 190 °C. The autoclaves were quickly loaded into the oven, and the timed heating began. The autoclaves were heated for different periods of time and were quenched in cold water. The pH of the liquid phase was measured. The liquid and solid phases of the product were separated by centrifugation (9500 rpm or 8475 g), and the solid phase was freeze-dried without further washing with water. The dried samples were sealed for subsequent characterization. The concentration of Al and P in the liquid phase was measured.

### Characterization

The NMR experiments were performed on a Varian Infinity-plus 400 spectrometer operating at a magnetic field strength of 9.4 T. The resonance frequencies at this field strength were 161.9 and 104.2 MHz for ^31^P and ^27^Al, respectively. A Chemagnetics 5 mm triple-resonance MAS probe with a spinning rate of 8 kHz was used to acquire the ^31^P and ^27^Al NMR spectra. The ^27^Al MAS spectra were acquired using a single pulse sequence with a short radio frequency (rf) pulse of 0.5 s (corresponding to a π/15 flip angle) and a pulse delay of 1.0 s. The pulse length for ^27^Al was measured using a 1 M Al(NO_3_)_3_ solution. The single-pulse ^31^P MAS NMR experiments with ^1^H decoupling were performed with a 90° pulse width of 4.6 ms, a 180 s recycle delay, and a ^1^H decoupling strength of 42 kHz. The chemical shifts were referenced to an 85% H_3_PO_4_ solution for ^31^P and a 1 M Al(NO_3_)_3_ solution for ^27^Al.

The powder X-ray diffraction (XRD) patterns were recorded on a Rigaku diffractometer that was equipped with a graphite monochromator using Cu Kα radiation (λ = 1.5418 Å). The pH values of the liquid phases were measured with a Sartorius PB-10 pH metre. The inductively coupled plasma atomic emission spectroscopy (ICP-AES) analysis was performed on a Perkin-Elmer Optima 3300DV spectrometer.

### Simulation

Dynamic calculations were performed at 433 K (160 °C) for APMeP150 and AP2pip and at 463 K (190 °C) for APMeP200 and AP2pip using Material Studio software^40^. Periodic boundary conditions (PBCs) were applied in one unit cell for each structure. The force field of DREIDING was adopted in the dynamic calculations^41^. The QEq method was used to equilibrate and redistribute the overall charge on the guest molecules and atoms of the framework^42^. Electrostatic interaction was evaluated through the Ewald summation method and van der Waals interactions with a 4.5 Å cut-off. The time step was set to 1.0 fs, and one million steps were run within the NVT ensemble during the dynamic calculation. The final single point energy of the non-bonding interaction between the inorganic host and the organic guest species was obtained at 0 K.

## Additional Information

**How to cite this article**: Huang, P. *et al.* Temperature-dependence of the influence of the position-2-methyl group on the structure-directing effect of piperazine in the synthesis of open-framework aluminophosphates. *Sci. Rep.* **6**, 22019; doi: 10.1038/srep22019 (2016).

## Supplementary Material

Supplementary Information

## Figures and Tables

**Table 1:** The non-bonding interactions for APMeP150, APMeP150 pip from MeP, AP2pip, and AP2pip MeP from pip.

Structure	Unit Cell Formula	Number of T in a Unit Cell	E_inter._/T (kJ/mol)
APMeP150	Al_6_P_8_O_32_·3(C_5_N_2_H_14_)	14	−81.91
APMeP150 pip from MeP	Al_6_P_8_O_32_·3(C_4_N_2_H_12_)	14	−45.64
AP2pip	Al_36_P_48_O_192_·18(C_4_N_2_H_12_)	84	−132.50
AP2pip MeP from pip	Al_36_P_48_O_192_·18(C_5_N_2_H_14_)	84	−60.59

**Table 2:** The non-bonding interactions of the APMeP200, AP2pip, and AP2pip MeP from pip.

Structure	Unit Cell Formula	Number of T in a Unit Cell	E_inter._/T (kJ/mol)
APMeP200	Al_36_P_48_O_192_·18(C_5_N_2_H_14_)	84	−126.03
AP2pip	Al_36_P_48_O_192_·18(C_4_N_2_H_12_)	84	−124.20
AP2pip MeP from pip	Al_36_P_48_O_192_·18(C_5_N_2_H_14_)	84	−116.17
