# Supplementary material for: Temperature-dependence of the influence of the position-2-methyl group on the structure-directing effect of piperazine in the synthesis of open-framework aluminophosphates
Source: Sci Rep. 2016 Feb 25;6:22019. doi: 10.1038/srep22019 (PMC4766482; doi:10.1038/srep22019)
Supplement: Supplementary Information [file srep22019-s1.pdf]

Supplementary Information

Temperature-dependence of the influence of the position-2-methyl group on the structure-directing effect of piperazine in the synthesis of open-framework aluminophosphates

Pai Huang,[1,3,#] Jun Xu,[2,#] Guodong Qi,[2] Feng Deng,[2] Ruren Xu[1] & Wenfu Yan[1*]

[1]State Key Laboratory of Inorganic Synthesis and Preparative Chemistry, College of Chemistry, Jilin University, 2699 Qianjin Street, Changchun 130012, P. R. China. [2]State Key Laboratory of Magnetic Resonance and Atomic and Molecular Physics, Wuhan Institute of Physics and Mathematics, The Chinese Academy of Sciences, Wuhan 430071, P. R. China. [3]Institute of Modern Agriculture, Jilin Economic Management Cadre College, 429 Guigu Street, Changchun 130012, P. R. China.

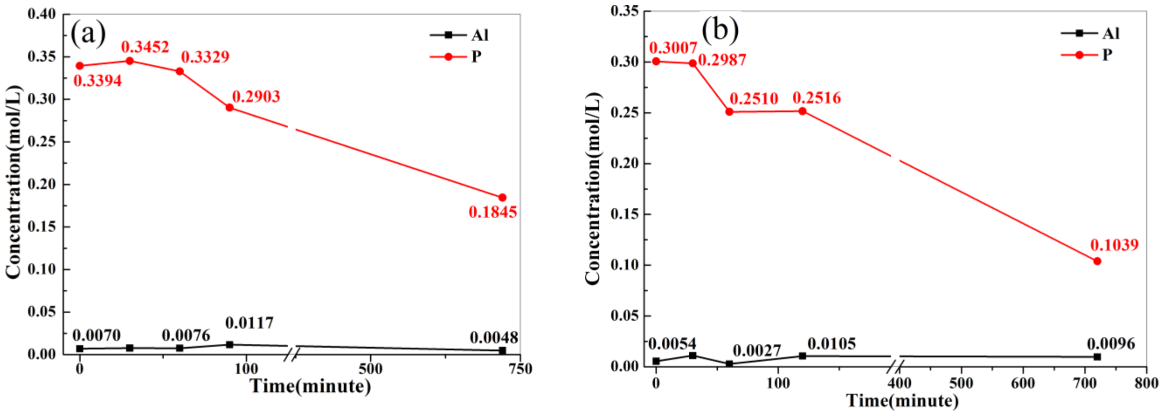

Figure S1 | The concentrations of Al and P in the liquid phase of the samples isolated throughout the hydrothermal treatment period during the APMeP150 (a) and AP2pip-160 (b) crystallization.

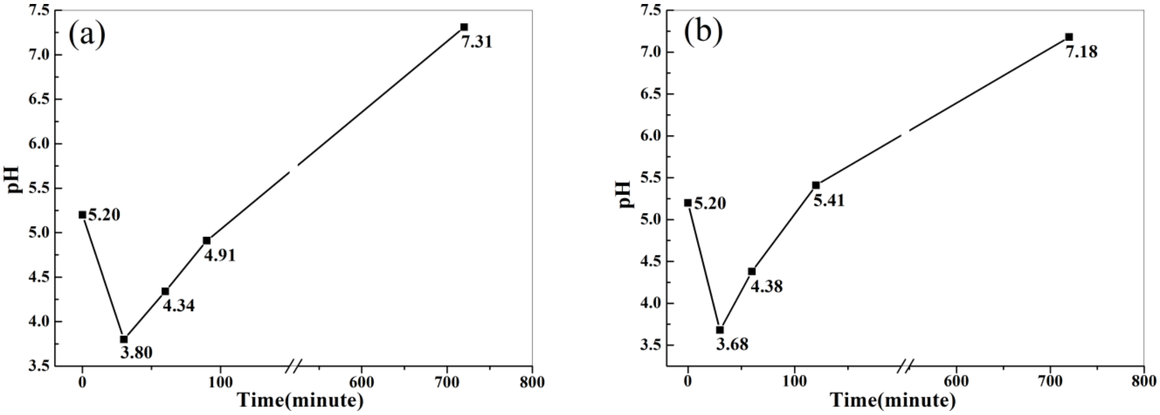

Figure S2 | The pH of the liquid phase of the samples isolated throughout the hydrothermal treatment period during APMeP150 (a) and AP2pip-160 (b) crystallization.

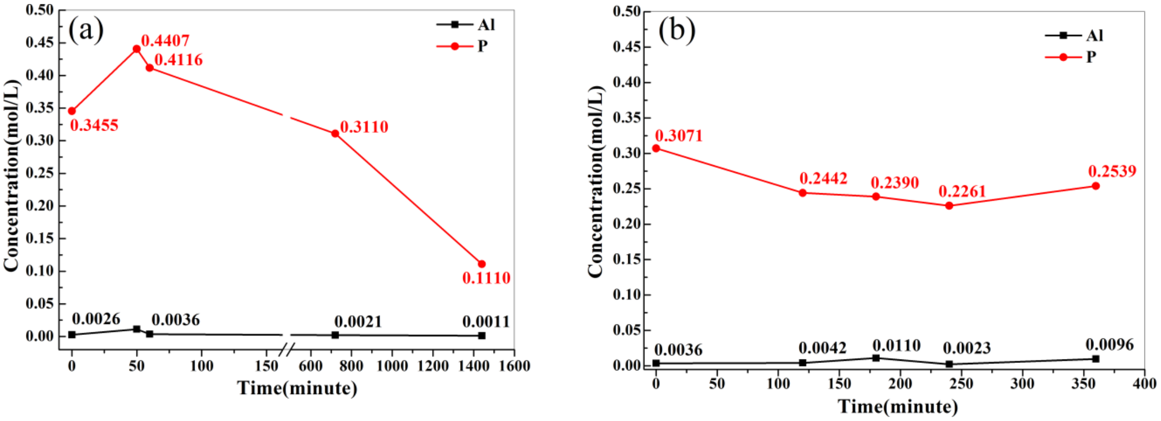

Figure S3 | The concentrations of Al and P in the liquid phase of the samples isolated throughout the hydrothermal treatment period during APMeP200 (a) and AP2pip-190 (b) crystallization.

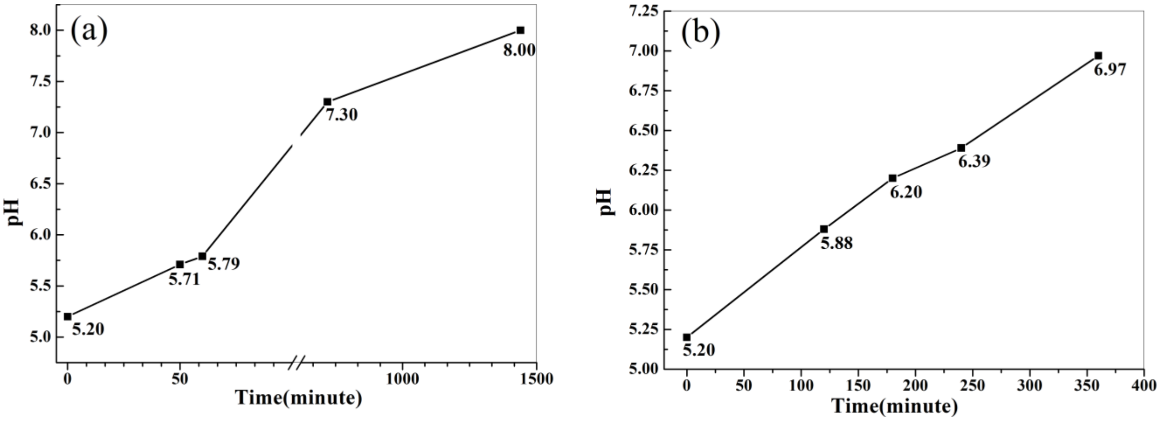

Figure S4 | The pH of the liquid phase of the samples isolated throughout the hydrothermal treatment period during APMeP200 (a) and AP2pip-190 (b) crystallization.

Table S1 | The bond lengths (Å) and angles (°) of four-coordinated Al in APMeP150 and AP2pip-160

APMeP150		AP2pip-160	
Al(2)-O(5)	1.6498	Al(7)-O(32)	1.6881
Al(2)-O(6)	1.7506	Al(7)-O(38)	1.6965
Al(2)-O(8)	1.7655	Al(7)-O(42)	1.7068
Al(2)-O(7)	1.7757	Al(7)-O(34)	1.7407
		Al(9)-O(40)	1.6908
		Al(9)-O(44)	1.6996
		Al(9)-O(48)	1.7107
		Al(9)-O(35)	1.7417
		Al(1)-O(45)	1.7018
		Al(1)-O(6)	1.7041
		Al(1)-O(3)	1.7107
		Al(1)-O(12)	1.7340
		Al(5)-O(20)	1.7067
		Al(5)-O(30)	1.7162
		Al(5)-O(28)	1.7182
		Al(5)-O(23)	1.7257
		Al(8)-O(2)	1.7087
		Al(8)-O(36)	1.7231
		Al(8)-O(39)	1.7297
		Al(8)-O(47)	1.7359
		Al(3)-O(18)	1.7096
		Al(3)-O(10)	1.7132
		Al(3)-O(14)	1.7143
		Al(3)-O(8)	1.7260

		Al(2)-O(11)	1.7099
		Al(2)-O(7)	1.7139
		Al(2)-O(15)	1.7149
		Al(2)-O(4)	1.7218
		Al(4)-O(24)	1.7219
		Al(4)-O(19)	1.7281
		Al(4)-O(26)	1.7300
		Al(4)-O(16)	1.7327
		Al(6)-O(43)	1.7230
		Al(6)-O(22)	1.7268
		Al(6)-O(31)	1.7321
		Al(6)-O(27)	1.7328
O(8)-Al(2)-O(6)	105.809	O(15)-Al(3)-O(4)	104.568
O(7)-Al(2)-O(8)	106.675	O(10)-Al(3)-O(14)	105.548
O(7)-Al(2)-O(6)	109.669	O(18)-Al(3)-O(10)	106.838
O(8)-Al(2)-O(5)	110.161	O(18)-Al(3)-O(14)	110.252
O(6)-Al(2)-O(5)	110.799	O(10)-Al(3)-O(8)	110.947
O(7)-Al(2)-O(5)	113.377	O(18)-Al(3)-O(8)	111.418
		O(11)-Al(2)-O(7)	105.235
		O(7)-Al(2)-O(4)	111.255
		O(11)-Al(2)-O(15)	111.821
		O(11)-Al(2)-O(4)	111.802
		O(7)-Al(2)-O(15)	112.33
		O(14)-Al(2)-O(8)	111.576
		O(40)-Al(9)-O(35)	105.403
		O(40)-Al(9)-O(48)	107.629

	O(44)-Al(9)-O(48)	108.985
	O(48)-Al(9)-O(35)	110.376
	O(44)-Al(9)-O(35)	111.296
	O(40)-Al(9)-O(44)	113.055
	O(2)-Al(8)-O(36)	105.585
	O(39)-Al(8)-O(47)	106.671
	O(36)-Al(8)-O(47)	110.037
	O(36)-Al(8)-O(39)	110.671
	O(2)-Al(8)-O(39)	110.906
	O(2)-Al(8)-O(47)	113.03
	O(22)-Al(6)-O(31)	106.46
	O(43)-Al(6)-O(27)	106.778
	O(31)-Al(6)-O(27)	109.958
	O(43)-Al(6)-O(22)	110.004
	O(43)-Al(6)-O(31)	111.307
	O(22)-Al(6)-O(27)	112.4
	O(24)-Al(4)-O(16)	106.573
	O(19)-Al(4)-O(26)	106.626
	O(24)-Al(4)-O(19)	110.566
	O(19)-Al(4)-O(16)	110.574
	O(26)-Al(4)-O(16)	110.886
	O(24)-Al(4)-O(26)	111.672
	O(32)-Al(7)-O(34)	107.411
	O(38)-Al(7)-O(42)	107.642
	O(32)-Al(7)-O(38)	108.998
	O(38)-Al(7)-O(34)	110.25
	O(42)-Al(7)-O(34)	111.057

	O(32)-Al(7)-O(42)	111.481
	O(12)-Al(1)-O(45)	107.458
	O(3)-Al(1)-O(45)	108.535
	O(6)-Al(1)-O(3)	108.867
	O(6)-Al(1)-O(45)	109.193
	O(6)-Al(1)-O(12)	109.834
	O(3)-Al(1)-O(12)	112.885
	O(28)-Al(5)-O(23)	108.533
	O(30)-Al(5)-O(28)	108.893
	O(20)-Al(5)-O(28)	109.427
	O(20)-Al(5)-O(30)	109.623
	O(30)-Al(5)-O(23)	109.661
	O(20)-Al(5)-O(23)	110.671
